# Tunable optical limiting optofluidic device filled with graphene oxide dispersion in ethanol

**DOI:** 10.1038/srep15362

**Published:** 2015-10-19

**Authors:** Chaolong Fang, Bo Dai, Ruijin Hong, Chunxian Tao, Qi Wang, Xu Wang, Dawei Zhang, Songlin Zhuang

**Affiliations:** 1 Engineering Research Center of Optical Instrument and System, the Ministry of Education, Shanghai Key Laboratory of Modern Optical System, University of Shanghai for Science and Technology, Shanghai, 200093, China; 2 The Institute of Photonics and Quantum Sciences, School of Engineering and Physical Sciences, Heriot-Watt University, Edinburgh EH14 4AS, UK

## Abstract

An optofluidic device with tunable optical limiting property is proposed and demonstrated. The optofluidic device is designed for adjusting the concentration of graphene oxide (GO) in the ethanol solution and fabricated by photolithography technique. By controlling the flow rate ratio of the injection, the concentration of GO can be precisely adjusted so that the optical nonlinearity can be changed. The nonlinear optical properties and dynamic excitation relaxation of the GO/ethanol solution are investigated by using Z-scan and pump-probe measurements in the femtosecond regime within the 1.5 μm telecom band. The GO/ethanol solution presents ultrafast recovery time. Besides, the optical limiting property is in proportion to the concentration of the solution. Thus, the threshold power and the saturated power of the optical limiting property can be simply and efficiently manipulated by controlling the flow rate ratio of the injection. Furthermore, the amplitude regeneration is demonstrated by employing the proposed optofluidic device. The signal quality of intensity-impaired femtosecond pulse is significantly improved. The optofluidic device is compact and has long interaction length of optical field and nonlinear material. Heat can be dissipated in the solution and nonlinear material is isolated from other optical components, efficiently avoiding thermal damage and mechanical damage.

Single-wall carbon nanotubes (CNT) and graphene have been demonstrated in many practical applications such as saturable absorption, optical switching and pulse regeneration owing to the extraordinary intrinsic properties including broadband nonlinearity and ultrafast recovery time^1^,^2^,^3^,^4^,^5^,^6^,^7^. As a derivative of graphene, graphene oxide (GO) is also an attractive nonlinear material, which has a monomolecular-layer structure with irregular spacing due to the oxidation process. GO presents various nonlinear properties, such as nonlinear refraction, nonlinear scattering, optical limiting and saturable absorption^8^,^9^. GO can be mass-produced by using the modified Hummers method at a low cost and is used as an intermediate for manufacturing graphene^10^,^11^. Moreover, the existence of carboxyl and hydroxyl groups makes GO hydrophilic. GO can be easily dissolved in liquids such as distilled water, ethanol, dimethylformamide (DMF), tetrahydrofuran (THF), and ethylene glyco^12^,^13^.

Optical limiting property makes the material transparent at a low input power but results in low transmission at a high input power. Devices of optical limiting property are capable of attenuating strong intensity of dangerous laser beams with an ultrafast response and exhibiting high transmission for low-intensity ambient light, so that the devices could be used in many applications, including eye and optical sensor protection from laser radiation, signal regeneration and power or energy regulation^14^,^15^,^16^. Thus, it attracts a lot of attention to study optical limiting property. Optical limiting property has been found in many graphene and GO based materials, such as graphene in N-methyl-2-pyrrolidone, N,N-dimethylacetamide, and γ-butyrolactone with 532 nm and 1064 nm nanosecond-regime excitation^5^, hydrogen exfoliated graphene with 532 nm nanosecond-regime excitation and 800 nm femtosecond-regime excitation^17^, graphene-porphyrin composite material with 532 nm nanosecond regime-excitation^18^, graphene-polyimide composite material with 532 nm nanosecond-regime excitation^19^, GO in DMF with 532 nm picosecond- and nanosecond-regime excitation^20^, alkyl-functionalized sub-stoichiometric GO in chlorobenzene, 1,2,4-trichlorobenzene, bromobenzene, 1,2-dichlorobenzene with 532 nm nanosecond-regime excitation^21^, GO nano-sheets and GO nano-ribbons with 532 nm and 1064 nm nanosecond-regime excitation^8^, GO nano-sheets and nano-ribbons doped in glass matrices with 532 nm picosecond- and nanosecond-regime excitation^22^,^23^, GO in distilled water with 800 nm femtosecond-regime excitation^24^, and GO impregnated polyvinyl alcohol sheet with 400 nm, 800 nm and 1400 nm femtosecond-regime excitation^25^. Usually, the investigations on optical limiting property of graphene and GO are carried out in the visible band or the near-infrared (NIR) band up to 1064 nm. Only few investigations are conducted in the 1.5 μm telecom band^26^,^27^. In addition, it was revealed that optical limiting property of GO was sensitive to oxygen functional groups^26^. By controlling the temperature in the hydrothermal dehydration process, oxygen functional groups could be removed from GO and tuning of optical limiting property was achieved. Nevertheless, the tuning process is accomplished during the manufacture of GO and cannot be simply realized in the practical use once the GO is produced.

Recently, optofluidic devices have drawn a lot of attention because the compatible association of microfluidics and photonics not only boost the development of microfluidic devices and systems by employing various highly precise and sophisticated optical fabrication technologies^28^,^29^,^30^ but also enrich the functions of the photonic devices by harnessing the advantages of microfluidic platforms including high sensitivity, reconfigurable capability and compactness^31^,^32^,^33^,^34^,^35^. Thanks to the combination of microfluidics and photonics, many optofluidic devices are developed with powerful tuning capability, such as tunable grating^36^, tunable diffraction grating^37^, variable optical attenuator^38^, tunable guided-mode resonance filter^39^ and tunable microlens^40^,^41^. Additionally, an in-fiber optofluidic device filled with CNT dispersion in DMF solvent was developed for passive mode-locking lasing^42^. It demonstrated an efficient and feasible approach to employ optofluidic device to exploit nonlinear optical (NLO) property of CNT.

In this work, we propose an optofluidic device filled with GO dispersion in ethanol. In the optofluidic device, the concentration of GO in the solution can be flexibly adjusted. By employing the optofluidic device, we analyze NLO properties, especially optical limiting property, of GO dispersion in the femtosecond regime within the 1550 nm telecom band. The influences of the concentration of GO dispersion over NLO properties are investigated. Since the optical limiting property is proportional to the concentration, we demonstrate the tunable optical limiting property of GO/ethanol solution. Furthermore, we use the proposed optofluidic device for pulse regeneration of an intensity-impaired signal.

## Results

### Optofluidic device

**Figure 1:**
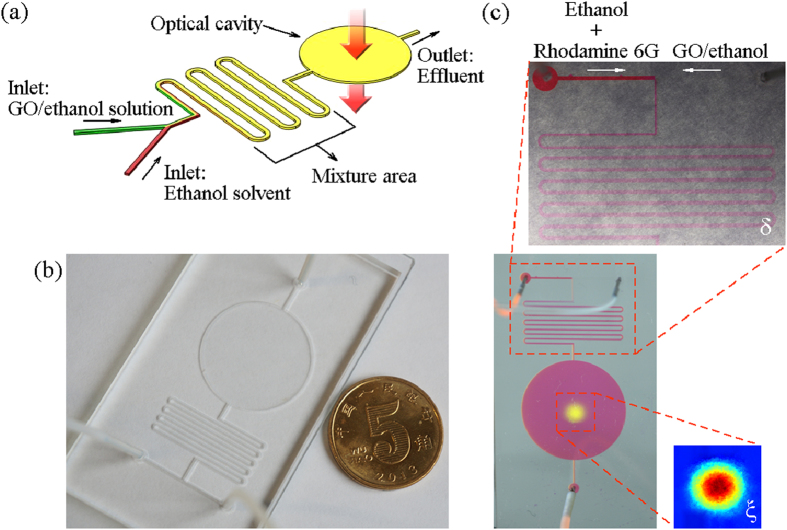
The Optofluidic device. (**a**) shows the schematic diagram of the design. It consists of two inlets, a zig-zag-shaped mixture area, an optical cavity to store the mixed solution and an outlet. (**b**) shows the fabricated optofluidic device. (**c**) shows the mixture of the GO/ethanol solution and ethanol solvent dyed with the Rhodamine 6G. Inset δ: The two liquids are completely mixed by using the zigzag geometry. Inset ξ: The reconstructed light spot of the emission distribution in the optical cavity.

The optofluidic device is designed as illustrated in Fig. 1(a). The device has two inlets, an outlet, a mixture area and an optical cavity. The prepared GO/ethanol solution and ethanol solvent are injected into the device from the two inlets separately with different flow rates. Then, the liquids are mixed in the mixture area. The mixture area has a zigzag geometry, because the zigzag design can aid in the mixture by changing the flow direction abruptly and increasing the length of the mixture area. Besides, since the density and viscosity of the liquids from the two inlets only have slight difference, the complete mixing can be easily achieved. After that, the optical cavity is used to store the completely mixed liquid. The light perpendicularly passes through the optical cavity. It is worth noting that the height of the optical cavity determines the interaction length of the optical field and the nonlinear material. The height of the optical cavity can be specially designed based on the requirement of the nonlinear effect. Finally, the effluent is output from the outlet.

The fabricated optofluidic device is shown in Fig. 1(b). The optofluidic device is fabricated in polydimethylsiloxane (PDMS) by photolithography technique. The PDMS is transparent within the 1550 nm band^43^. The fabrication of the optofluidic device is simple and at a low cost. The width and the height of the channel are 200 μm and 100 μm, respectively. The total length of the channel in the mixture area is about 180 mm, which is long enough for complete mixing. The radius of the optical cavity is 10 mm.

**Figure 2:**
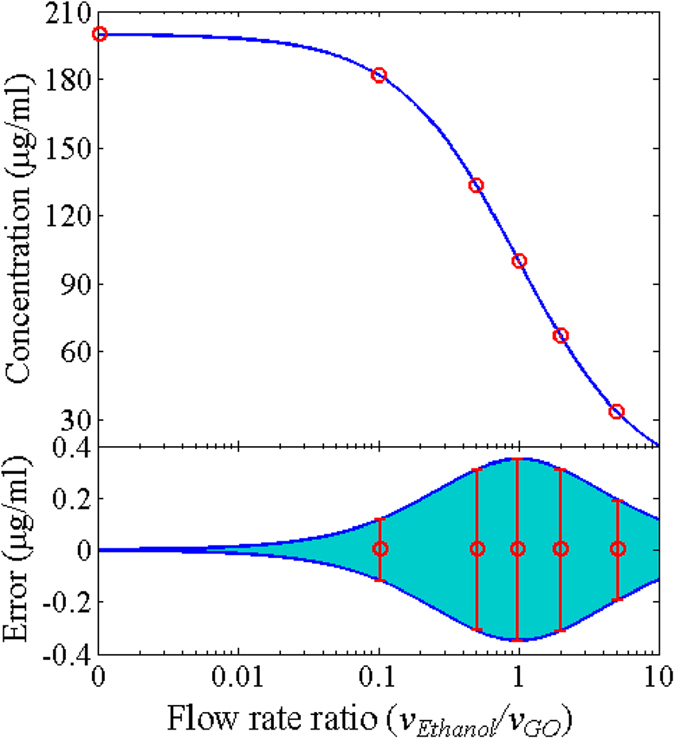
Characteristics of the optofluidic device. The concentration and error of the mixed solution in the optical cavity versus the flow rate ratio of the injections. Blue solid lines: the calculated concentration and error. Red circles: the concentration used in the experiment. The deviation of the concentration is calculated based on the flow accuracy of the syringe pumps.

Due to the existence of hydroxyl and carboxyl, GO presents hydrophilic characteristic and can be dissolved in various liquids including distilled water, ethanol, DMF and THF. In the experiment, 99.5% ethanol is selected as solvent because ethanol is non-toxic and has low viscosity. 200 μg/ml GO/ethanol solution is prepared. The color of the solution is dark yellow and no agglomeration can be observed. The prepared GO/ethanol solution and 99.5% ethanol solvent are injected into the optical device by using two syringe pumps (Harvard Apparatus PHD 2000). The concentration of the mixed GO/ethanol solution in the optical cavity is determined by the flow rate ratio of the injection, which can be calculated as follows.


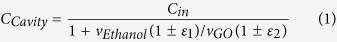


where *C*_*in*_ is the concentration of the prepared GO/ethanol solution injected into the device, *v*_*GO*_ and *v*_*Ethanol*_ are the flow rates of the prepared GO/ethanol solution and ethanol solvent, and ε_1_ and ε_2_ are the flow accuracy of the two syringe pumps. Figure 2 depicts the relationship between the concentration of the mixed GO/ethanol solution in the optical cavity and the ratio of the flow rates of the two injected liquids. The concentration of the mixed GO/ethanol solution drops dramatically with the increment of the flow rate of the ethanol solvent when the flow rate of the prepared GO/ethanol solution is fixed. Thus, the change of the concentration of GO in the optical cavity is very flexible. The deviation of the concentration is resulted from the flow accuracy of the syringe pumps. The syringe pumps used in the experiment has the flow accuracy of 0.35%. The calculated deviation of the concentration is also shown in Fig. 2. The deviation is no more than ±0.4 μg/ml, which is rather small comparing to the required concentration.

In order to observe the mixture of the liquids, fluorescent dye Rhodamine 6G (excitation wavelength of 526 nm and emission wavelength of 560 nm) is dissolved into the ethanol solvent which then becomes red. A CMOS camera is used to monitor the mixture. The flow rates of the GO/ethanol solution and the dyed ethanol solvent are 100 μl/s and 50 μl/s. In Fig. 1(c), it shows that the whole channel turns into reddish color after the fourth bend and the color becomes lighter, which hints that the two liquids can be completely mixed before entering the optical cavity. To further investigate the mixing performance and the uniformity of the solution in the optical cavity, a 532 nm diode-pumped solid-state laser is used to illuminate the optical cavity. The light beam from the laser has a Gaussian shape. When the light illuminates the optical cavity, the light beam is visualized by Rhodamine 6G and a bright yellow spot can be clearly observed. The light spot presents a Gaussian-shaped distribution and there is no abrupt change in the intensity of the emission distribution, indicating that the two liquids are completely mixed and the solution in the optical cavity is uniform.

### Characteristics of GO dispersion

GO dispersion plays an important role in the proposed device, because GO exhibits attractive NLO properties. Open aperture Z-scan technique, which is a powerful technique to characterize the optical limiting properties, is used to measure NLO properties of the GO/ethanol solution in the femtosecond regime within the 1.5 μm band. A home-made femtosecond laser is employed to generate 80 fs (full-width at half-maximum) optical pulses with the wavelength center of 1561 nm. The repetition rate is 1 MHz and the average power is about 55 mW. The optofluidic device is placed in between a pair of biconvex lenses of 50 mm focal length. The laser beam is focused by the first biconvex lens. At the focal point, the beam waist is 13.8 μm (the radius of the light spot when the intensity falls to *1/e*^*2*^). The Rayleigh length, 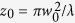, where *w*_*0*_ is the beam waist radius at focus and *λ* is the wavelength of light, is 383 μm. Since the Rayleigh length is larger than the height of the optical cavity, the GO dispersion within the optical cavity can be regarded as ‘thin’ sample, satisfying the condition for Z-scan technique. The optofluidic device is moved around the focal point along the z-axis to experience the variation of the incident intensity. After the second lens, the power is measured by a detector.

To investigate the influence of the concentration over NLO properties, the ratio of the injection flow rates is changed. The prepared GO/ethanol solution is injected into the device at a fixed flow rate of 20 μl/s, while the ethanol solvent is injected via the other inlet at the flow rate of 0 μl/s, 2 μl/s, 10 μl/s, 20 μl/s, 40 μl/s or 100 μl/s. The corresponding concentration of the mixed solution in the optical cavity is 200 μg/ml, 181.81 μg/ml, 133.3 μg/ml, 100 μg/ml, 66.67 μg/ml and 33.33 μg/ml, respectively, as marked with red circles in Fig. 2.

**Figure 3:**
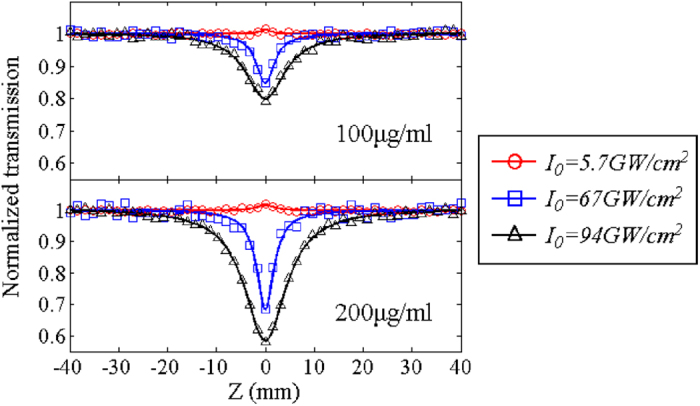
Open aperture Z-scan results of the GO/ethanol solution with the concentration of 100 μg/ml and 200 μg/ml. Solid lines represent the theoretical fits.

Figure 3 illustrates open-aperture Z-scan measurement results for 100 μg/ml and 200 μg/ml GO/ethanol solution. When the incident intensity is low, i.e. the intensity of the light at focus *I*_*0 *_= 5.7 GW/cm^2^, small peaks can be observed, indicating that saturable absorption (SA) occurs. However, in the Z-scan measurement, SA is not obvious even at a very low incident power. The existence of SA is explained as that the absorption cross section in the excited state is lower than that in the ground state and thus the transmission is enhanced when the GO is strongly excited^20^. With the increase of the incident intensity, i.e. *I*_*0 *_= 67 and 94 GW/cm^2^, valleys appear at the focal point, implying that optical limiting happens. Optical limiting may be resulted from two main mechanisms, nonlinear absorption and nonlinear scattering. Reverse saturable absorption (RSA) and two photon absorption (TPA) are two main mechanisms contributing to nonlinear absorption. Similarly to SA, RSA originates from excited state absorption (ESA) process. RSA happens when the absorption cross section in the excited state is higher than that in the ground state and thus the transmission becomes weak with the increase of the incident intensity. ESA is a dominant mechanism for resonant and near resonant excitations, while TPA dominates the nonlinear absorption for non-resonant excitation. It was found that the contribution of TPA to nonlinear absorption was important in the short-pulse regime^20^. In addition, it is known that nonlinear scattering also leads to optical limiting of GO with 532 and 1064 nm laser excitation^5^,^44^. According to Mie scattering theory, the scattering occurs when the scattering centers have the size on the order of the wavelength of the incident light. Thus, GO sheets of several hundred nanometers cause scattering. Besides, if the incident intensity is high, the light heats GO sheets and transfers the thermal energy to the surrounding solution, evaporating the solution and producing gas bubbles. The bubbles cause scattering as well. To investigate the influence of nonlinear scattering, scattered light is measured but it is not obvious. The findings are in agreement with the previous research^27^, which revealed that in the NIR regime nonlinear scattering has no significant contribution to the optical limiting of GO and nonlinear absorption is a dominant factor.

**Figure 4:**
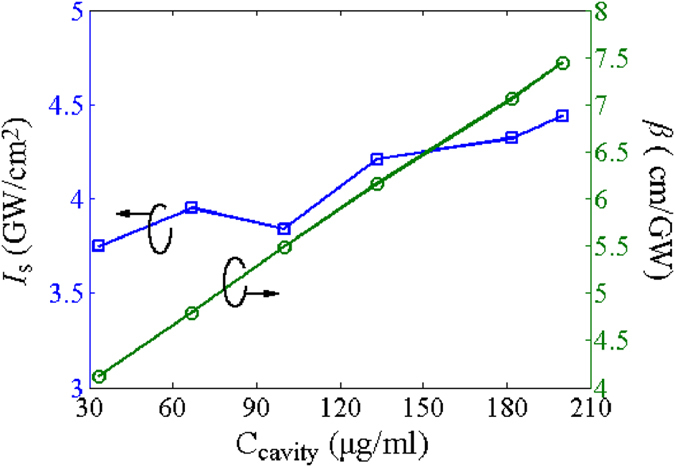


The absorption coefficient can be characterized as 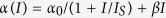, where *α*_*0*_ is the linear absorption coefficient, which is 2.1 × 10^4 ^cm^−1^ and *I*_*S*_ is the saturation intensity, respectively. *β* is the sum of contribution from nonlinear absorption and nonlinear scattering. For a temporally Gaussian pulse, the normalized transmittance can expressed as


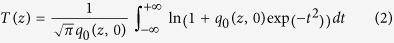


where 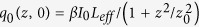. *I*_*0*_ is the peak intensity at the focal point. *L*_*eff*_ is the effective length of sample, i.e. 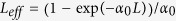. *L* is the thickness of the sample, i.e. the height of the optical cavity. The intensity variation along the propagation direction can be can be described as 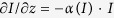. Then, by fitting the normalized transmittance with the open-aperture Z-scan measurement results, *I*_*s*_ and *β* can be unambiguously deduced, as shown in Fig. 4. Saturation intensity is not closely related to the concentration, but the contribution of nonlinear absorption and nonlinear scattering are concentration-dependent, which agrees with the findings in the nanosecond and picosecond regimes at 532 nm and 1064 nm^45^. *β* becomes larger with the concentration of GO/ethanol solution, which indicates that the contribution of nonlinear absorption and nonlinear scattering are enhanced.

**Figure 5:**
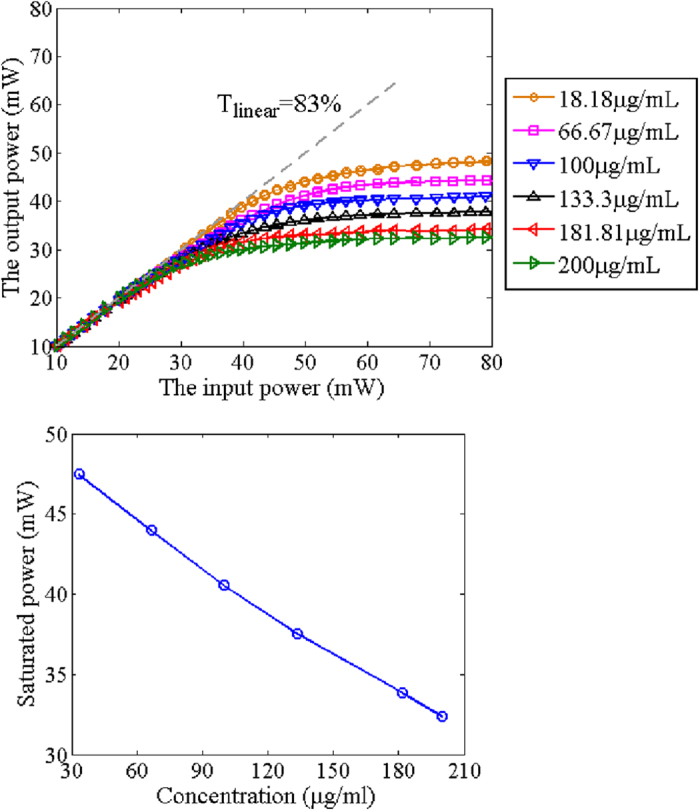
Normalized transient transmission *ΔT/T* dependent relaxation dynamics of the GO/ethanol solution with the concentration of 100 μg/ml and 200 μg/ml. The relaxation dynamics is measured by femtosecond pump-probe technique. Solid lines are the fits to a bi-exponential decaying function, *ΔT/T = A*_*1*_* exp(-t/τ*_*1*_*) + A*_*2*_* exp(-t/τ*_*2*_), convolved with the pump and probe pulse profiles.

The relaxation dynamics of the GO/ethanol solution with different concentrations is investigated by using the degenerate pump-probe measurement. The pump pulse and probe pulse have the intensity of 71 GW/cm^2^ and 6.2 GW/cm^2^, respectively. The measured transient differential transmission, *ΔT/T*, for 100 μg/ml and 200 μg/ml GO/ethanol solution is shown in Fig. 5. Δ*T/T* represents the difference between the transmission of the probe with and without the pump excitation, normalized by the transmission of the probe without the pump excitation. After the pump excitation, the transmission abruptly drops due to the photo-induced nonlinear absorption and then rapidly recovers. The recovery time is attributed to carrier-carrier scattering and carrier-acoustic phonon scattering, corresponding to fast and slow time constants, *τ*_*1*_ and *τ*_*2*_^25^,^46^. Therefore, the measured data is fitted by using a bi-exponential decaying function convolved with the pump and probe pulse profiles. The fast time constants are 245 fs (65%) and 265 fs (60%) for the concentration of 100 μg/ml and 200 μg/ml and the slow time constants are 3.5 ps (35%) and 3.55 ps (40%), respectively. The relaxation dynamics is dominated by the fast time constant. According to the analysis of pump-probe measurement results, it is found that the GO/ethanol solution presents ultrafast recovery time and the recovery time is not affected by the concentration.

### Tunable optical limiting property

The GO/ethanol solution presents significant NLO properties and fast recovery time. Particularly, the optical limiting property is closely related to the concentration of the solution. An experimental demonstration on the tuning of the optical limiting property is conducted in the femtosecond regime within the 1550 nm telecom band. In the experiment, a passively mode-locked erbium-doped fiber femtosecond laser (MenloSystems T-Light) is used. The laser has the repetition rate of 100 MHz and the pulse width (FWHM) is 90 fs. The average power of the laser is about 130 mW. The center wavelength of the laser is 1557 nm. The light from the laser perpendicularly passes through and focuses in the optical cavity of the optofluidic device via a power attenuator, a collimator, a beam expander and a biconvex lens. The beam waist at focus is about 1.6 μm. Then, the light is collected by using another set of biconvex lens, beam expander and collimator, and measured by using a power meter.

**Figure 6:**
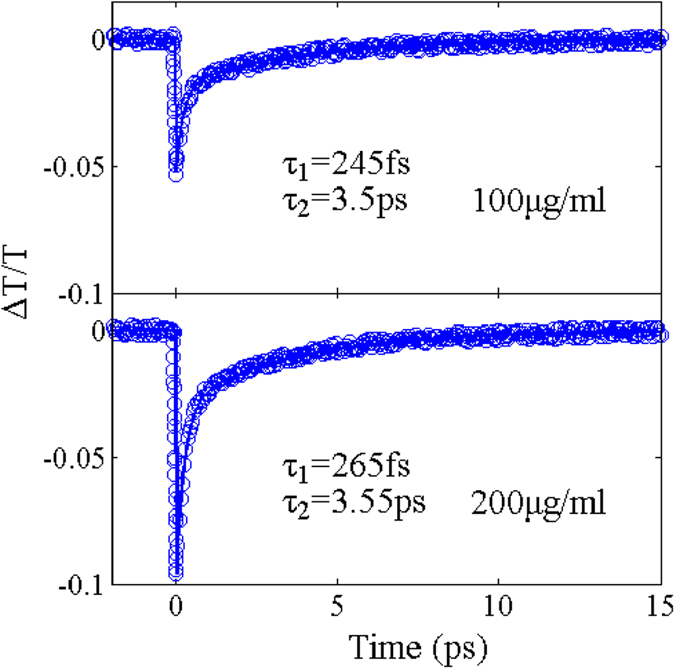
The measured optical limiting property. (**a**) shows the influence of the concentration of GO/ethanol solution over the optical limiting property. *T*_*linear*_ is the linear transmission. (**b**) shows the relationship of the concentration of GO/ethanol solution and the saturated power.

Figure 6(a) shows the measured power transfer function for the different concentrations of the GO/ethanol solution. The linear transmission, *T*_*linear*_, is 83%. When the input average power is low, the device operates in the unsaturated regime. The output power linearly increases with the increment of the input power. By increasing the input power, the device is heavily saturated. The output power becomes almost constant and the device operates as an optical limiter. Additionally, if the concentration of GO is high, the device gets saturated at a relatively low input power, i.e. low threshold power, and thus the saturated power is low. Figure 6(b) shows the relationship between the concentration of the GO/ethanol solution in the optical cavity and the saturated power which is measured when the input power is at 70 mW. The saturated power decreases with the increase of the concentration, because the GO/ethanol solution with high concentration contributes to strong nonlinear absorption. Thus, there is a compromise between the output intensity and the dynamic control range of the input power. The optofluidic device offers a simple and flexible way to control the optical limiting property so that it is straightforward to meet the requirements of various applications.

### Amplitude regeneration

**Figure 7:**
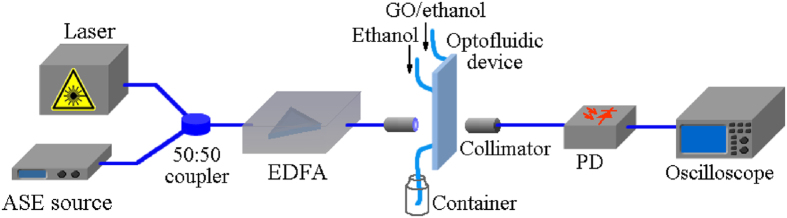
Schematic of the experimental setup. A train of femtosecond optical pulses are firstly coupled with ASE noise by using a 50:50 optical coupler. An EDFA is then used to compensate the power loss of the coupling. A pair of collimators is used to couple the light into the free space and back to the fiber and the optofluidic device is place in between. Finally, an oscilloscope is to measure the waveform. ASE source: amplified spontaneous emission sourse. EDFA: erbium-doped fiber amplifier. PD: photodetector. This figure was drawn by Bo Dai.

To further investigate the optical limiting property of the proposed optofluidic device, an experiment of amplitude regeneration is carried out. In the signal regeneration, it requires to attenuate high-intensity signal while keep the low-intensity signal as the input. The threshold power of optical limiting should be around the low-intensity signal so as to guarantee high power efficiency. Figure 7 shows the experimental setup. A pulse train generated from the femtosecond laser is coupled with amplified spontaneous emission (ASE) noise by an optical coupler. An erbium-doped fiber amplifier (EDFA) is followed to compensate the loss of the coupling. Then, the light passes through the optical cavity of the optofluidic device and is fed into a photodetector for optical-to-electrical conversion via a pair of collimators. Finally, the waveform of the signal is measured by using an optical sampling oscilloscope.

**Figure 8:**
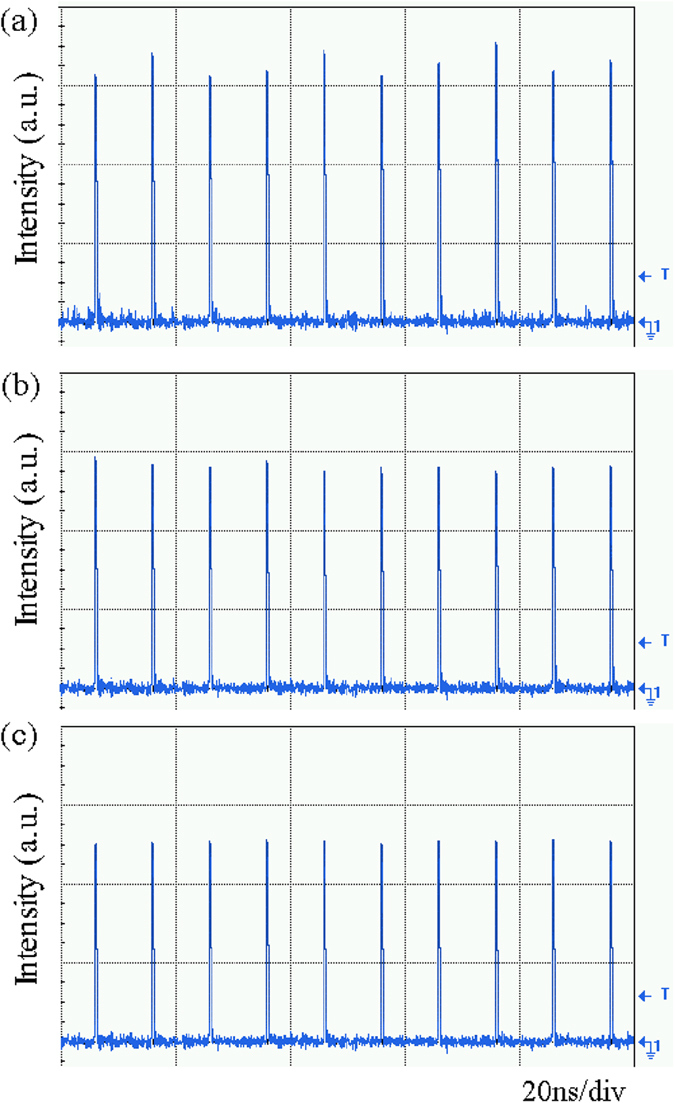
The measured waveforms without and with amplitude regeneration. (**a**) shows the intensity-impaired waveform. (**b**) and (**c**) show the measured waveforms after amplitude regeneration when the optofluidic device is filled with the GO/ethanol solution of the concentration of 66.67 μg/ml and 200 μg/ml, respectively.

When the optical cavity is empty, the signal is intensity-impaired, as shown in Fig. 8(a). The average power is about 58 mW. The fluctuation of the optical pulses is severe and the deviation of the peak intensity is 21.2%, indicating a low signal quality. Then, the 99.5% ethanol solvent and the prepared 200 μg/ml GO/ethanol solution are injected into the device simultaneously with the flow rate ratio of 2:1, resulting in that the concentration of the mixed solution in the optical cavity is 66.67 μg/ml. The measured waveform is shown in Fig. 8(b). Only slight fluctuation of can be observed. The deviation of the peak intensity becomes 7.9%. To achieve a better performance, the GO/ethanol solution of high concentration is preferred. According to the power transfer function of the device, the GO/ethanol solution of 200 μg/ml concentration is used. The prepared GO/ethanol solution is injected into the device and meanwhile the injection of the ethanol solvent is stopped. The output waveform is measured as shown in Fig. 8(c). The fluctuation of the optical pulses is efficiently suppressed. The deviation of the peak intensity is reduced to 2.4%. The signal quality is significantly improved. It proves the feasibility of the amplitude regeneration by using the proposed device. With the increase of the concentration of GO/ethanol solution in the optical cavity, the signal quality becomes better, but the output power drops. In the practical use, optical limiting should be tuned to a certain level where the tradeoff between the tolerable performance and the high output power can be balanced. The tuning of the flow rate ratio of the injection is capable of adjusting the threshold power and the saturated power, which makes the device suitable for other applications.

## Discussion

We have proposed an optofluidic device which can precisely control the concentration of the GO/ethanol solution. In the investigation of the GO/ethanol solution, we have carried out the Z-scan and pump-probe measurements to characterize NLO properties and relaxation dynamics. The GO/ethanol solution presents strong NLO properties, especially optical limiting property, and has ultrafast recovery time. The optical limiting property is proportional to the concentration of the solution, while relaxation dynamics is not affected by the concentration change. Furthermore, the tuning of optical limiting property is studied in detail. The feasibility of the tunable optical limiting property has been proved in the femtosecond regime within the 1550 nm telecom band. The threshold power and the saturated power can be simply and flexibly changed by adjusting the flow rate ratio of the injection. It is worth noting that there is a tradeoff between the output power and the dynamic range of the input power. In addition, the amplitude regeneration has been demonstrated. By using the proposed optofluidic device, the signal quality can be significantly improved without changing the input power.

Comparing to the configurations that CNT or graphene/GO is deposited on a fiber ferrule or coated on the surfaces of a D-shaped fiber or a tapered fiber, the proposed configuration using optofluidic device has many advantages. The three-dimensional structure allows the optical field to interact with the nonlinear material directly and along a relatively long interaction length. Besides, the peak power of the input optical pulse was more than 95 GW/cm^2^ and the device operated well. The thermal damage resulted from the high optical power to the nonlinear material can be efficiently overcome, because the nonlinear material is dispersed in the solvent and heat dissipation can be realized. In addition, the optofluidic device can be easily inserted in the optical path without contacting with the fibers, avoiding mechanical damage. The proposed cost-efficient optofluidic device was flexible in the function and stable in the operation, which demonstrated a fantastic combination between microfluidics and photonics.

## Methods

### Preparation of GO dispersion

The nonlinear material, GO, is used as the additive in the solution and it is prepared by the modified Hummers method. The procedure of GO preparation is briefly introduced as follows. Firstly, 2 g graphite power and 1 g sodium nitrate are added into the icy 60 ml 98% sulfuric acid. The mixture is placed in an ice bath for 30 mins. Then, 6 g potassium permanganate is gradually added to the mixture. The mixture is under mild agitation for 30 mins, and meanwhile the temperature is kept below 10 °C. After that, the mixture is warmed to 40 °C for 30 mins. 60 ml deionized water is added to the mixture and the temperature is increased to 95 °C. After 45 mins, the mixture is diluted to 300 ml. To oxidize the graphite, 15 ml 30% H_2_O_2_ is added to the mixture. The color of the solution is turned to yellow and it means that the oxidation of graphite is realized. The graphite oxide is placed in the ultrasonic generator. During the ultrasonication, the GO sheets are exfoliated from the graphite oxide. Then, 20 ml 5% hydrogen chloride and distilled water are used to wash the GO sheets. The centrifugation is conducted at the rate of 10000 rpm for several times. The GO sheets are dried at 50 °C. Before preparing the GO/ethanol solution, the structure, defect and disorder of the home-made GO are characterized by measuring the Raman spectrum of the GO. The Raman spectrum is measured under the excitation of the GO with a 532 nm light. There are two main high peaks (D and G peaks) at 1352 cm^–1^, and 1600 cm^–1^, and a very low peak (2D peak) at 2937 cm^–1^, respectively. The D peak is from the in-plane bond stretching of *sp*^2^ carbon atoms, while the G peak arises from the defect and disorder in the structure due to the attachment of hydroxyl, carboxyl and epoxide groups, destroying the bonds on the carbon basal plane. The 2D peak reveals the existence of the graphene and the low level of 2D peak indicates that the oxidation of graphite is achieved well. Finally, 32 mg GO sheets are added into the 160 ml 99.5% ethanol to prepare 200 μg/ml GO/ethanol solution, and the solution is placed in the ultrasonic generator for 1 hour.

## Additional Information

**How to cite this article**: Fang, C. *et al.* Tunable optical limiting optofluidic device filled with graphene oxide dispersion in ethanol. *Sci. Rep.* **5**, 15362; doi: 10.1038/srep15362 (2015).
